# Volatile Organic Compound Gamma-Butyrolactone Released upon Herpes Simplex Virus Type -1 Acute Infection Modulated Membrane Potential and Repressed Viral Infection in Human Neuron-Like Cells

**DOI:** 10.1371/journal.pone.0161119

**Published:** 2016-08-18

**Authors:** Kevin Rochford, Feng Chen, Yan Waguespack, Robert W. Figliozzi, Madan K. Kharel, Qiaojuan Zhang, Miguel Martin-Caraballo, S. Victor Hsia

**Affiliations:** 1 Department of Pharmaceutical Sciences, School of Pharmacy and Health Professions, University of Maryland, Eastern Shore, Princess Anne, Maryland, 21853, United States of America; 2 Department of Natural Sciences, School of Agricultural and Natural Sciences, University of Maryland, Eastern Shore, Princess Anne, Maryland, 21853, United States of America; Cincinnati Children's Hospital Medical Center, UNITED STATES

## Abstract

Herpes Simplex Virus Type -1 (HSV-1) infections can cause serious complications such as keratitis and encephalitis. The goal of this study was to identify any changes in the concentrations of volatile organic compounds (VOCs) produced during HSV-1 infection of epithelial cells that could potentially be used as an indicator of a response to stress. An additional objective was to study if any VOCs released from acute epithelial infection may influence subsequent neuronal infection to facilitate latency. To investigate these hypotheses, Vero cells were infected with HSV-1 and the emission of VOCs was analyzed using two-dimensional gas chromatograph/mass spectrometry (2D GC/MS). It was observed that the concentrations of gamma-butyrolactone (GBL) in particular changed significantly after a 24-hour infection. Since HSV-1 may establish latency in neurons after the acute infection, GBL was tested to determine if it exerts neuronal regulation of infection. The results indicated that GBL altered the resting membrane potential of differentiated LNCaP cells and promoted a non-permissive state of HSV-1 infection by repressing viral replication. These observations may provide useful clues towards understanding the complex signaling pathways that occur during the HSV-1 primary infection and establishment of viral latency.

## Introduction

Herpes Simplex Virus Type -1 (HSV-1) is one of the most common causes of infectious disease in humans, and its early detection could avoid serious complications such as encephalitis, and further spread of the virus. After the initial infection, HSV-1 may establish latency, with reactivation occurring transitorily, causing another round of lytic assaults, making its early detection a challenge [1]. Usually the viral infection results in mild symptoms (or is asymptomatic) resulting in the near (or complete) absence of indicators as to the presence of infection. However, the HSV-1 infection may trigger serious complications such as keratitis or encephalitis [2], and is typically revealed by the manifestation of cytopathic effects such as dendritic ulcers or necrosis, altering cellular metabolism. Similar to other herpes viruses, it could launch latent infection in patients by maintaining a dormant state in the sensory neurons of trigeminal ganglia (TG) [1]. The molecular mechanisms involved in the establishment of viral latency are not clearly understood. Given that a HSV-1 infection, like a bacterial infection, may produce volatile organic compounds (VOCs) that would provide a unique identifier as to its presence, it merits exploring whether such VOC signatures exist, that can then be exploited in the development of a medical test for the early detection of HSV-1. The measurement and analysis of VOCs produced during a HSV-1 infection appears yet to have been undertaken.

**Fig 1:**
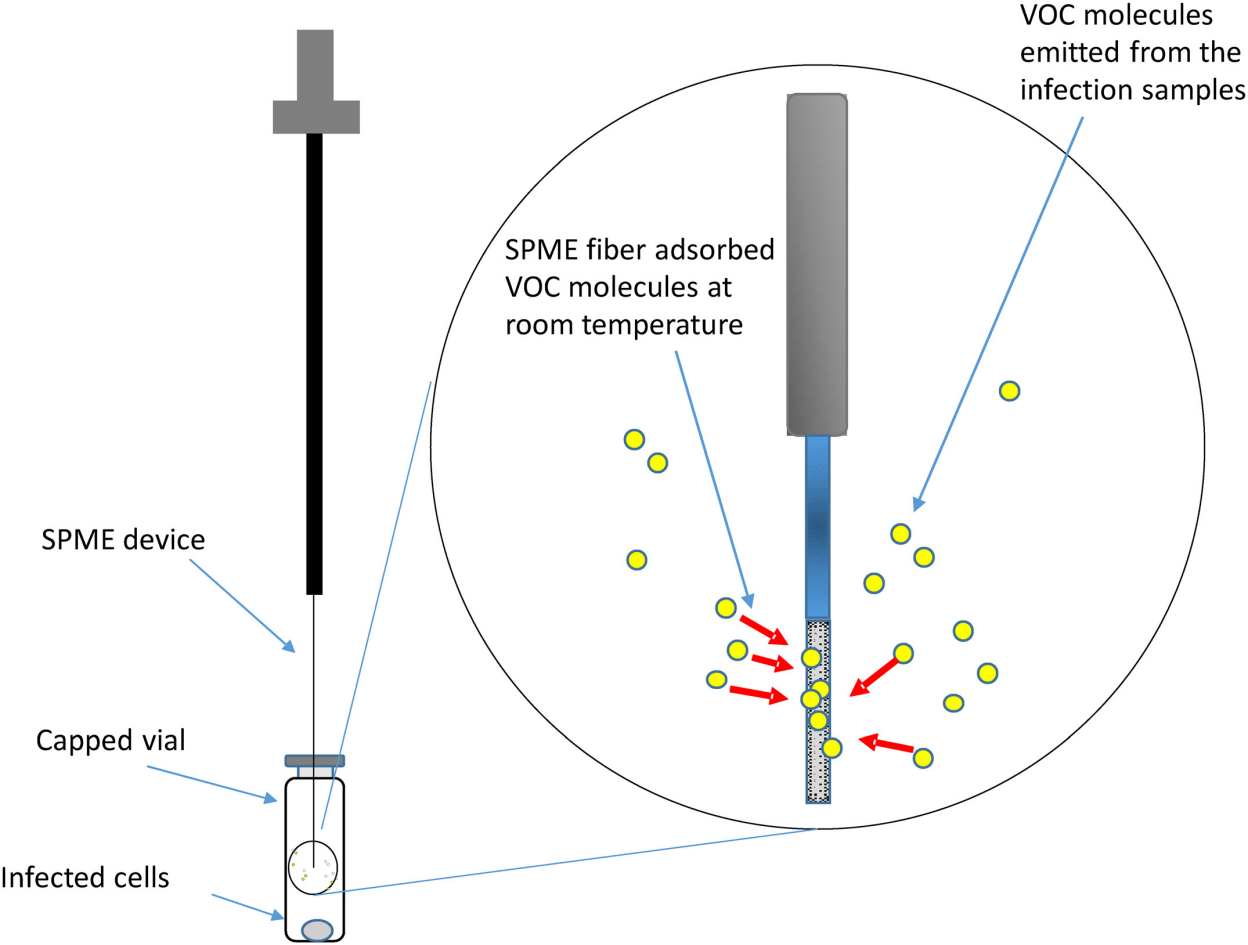
Experimental Design of VOC detection by GC-MS. HSV-1 infected cells were collected and stored in capped vials, with a SPME device inserted into the headspace of the vial to adsorb the VOC molecules emitted from the infected samples. The adsorbed VOCs were released under high temperature at 240°C at the GC inlet for subsequent GC-MS analyses.

In this project, the changes in VOC concentrations caused by HSV-1 acute infection were analyzed using two-dimensional gas chromatograph/mass spectrometry (2D GC/MS) with the goal of identifying potential indicators that could be exploited for unique detection of this type of infection. The infection of cells was conducted for different time periods, and the VOCs from the infected cells were collected via headspace sampling using a 50/30 μm Divinylbenzene/Carboxen Solid Phase MicroExtraction (SPME) device (Fig 1A) for subsequent 2D GC/MS analysis. The latter captured changes in concentrations of a number of VOCs, including a significant increase in gamma butyrolactone (GBL) produced by HSV-1 infected cells that became more substantial with increasing infection time. Furthermore, the GBL appeared to modulate the resting membrane potential (RMP) of tested differentiated cells as well as regulate viral replication.

## Materials and Methods

### Cell lines and viruses

The Vero cells (Cat#: ATCC^®^ CCL-81^™^) were grown in Dulbecco’s Modified Eagle Medium (DMEM) supplemented with 10% Fetal bovine serum (FBS). The cells were grown and maintained at 37°C and 5% CO_2_ in a cell culture incubator. Lymph Node Carcinoma of the Prostate (LNCaP) cells were purchased from ATCC (Cat#: CRL-1740) and cultured in a Roswell Park Memorial Institute medium (RPMI -1640) with 10% FBS. For neuronal differentiation, LNCaP cells were cultured at a density of 4×10^3^ cells/cm^2^ of growth area, with differentiation being triggered by androgen deprivation as previously described [3]. The HSV-1 strain 17-Syn^+^/GFP was used for this experiment and the expression of its green fluorescent protein (GFP) exploited for detection of the infection [4]. The differentiated LNCaP cells exhibited neuronal morphology/physiology and were used for HSV-1 neuronal infection as previously described [3].

The infection of Vero and LNCaP cells was performed by first removing the media, then adding 200 μL of serum free media containing the virus at a multiplicity of infection (moi) of 1. Inoculates were left in the cultures for one hour for viral attachment and entry. Afterwards, the inoculums were replaced with fresh culture media and incubated for 1, 5, 10, and 24 hours. Infected cell monolayers were then harvested into 5 mL samples using trypsin. They were transferred to a glass bottle with a septum cap and then stored in a -20°C refrigerator before headspace sampling (Fig 1). The media removed during cell extraction was also collected and subjected to headspace sampling followed by GC-MS analyses.

### SPME headspace sampling

The Solid Phase MicroExtraction (SPME) apparatus and fibers (50/30 μm Divinylbenzene/Carboxen^™^ on polydimethylsiloxane coating) used for headspace sampling were obtained from Supelco (Bellefonte, PA). Prior to use, each new fiber was conditioned by heating it at 240°C for 2 hours. Headspace sampling was performed by inserting the syringe needle of the SPME assembly through the septum cap into the headspace above the samples, cell pellet, or media (Fig 1), and extending the fiber through the syringe needle into the headspace to allow the volatiles to be adsorbed into the fiber (Fig 1A). After 12 hours of adsorption time at room temperature to allow for a VOC absorption equilibrium to be established, the fiber was withdrawn into the outer septum piercing needle, removed from the vial, and subsequently thermally desorbed in the heated injection port of the gas chromatograph (GC) at 240°C for 5 minutes. To remove any remaining analytes from the stationary phase of the fiber, the fibers were heated at 240°C for 30 minutes before each measurement.

### GC/MS measurement and analyses

Analysis of the VOCs of the infected Vero cell samples for different infection times, uninfected Vero Cell sample (control), trypsin, and media were all carried out using a 2D GC/MS system comprised of an Agilent 7860 GC (Wilmington, DE) with two sequential columns and an Agilent 5975 MS (Wilmington, DE). The first column was an SGE BPX-5 (Austin, TX) column utilizing boiling points for separation, and the second column was an SGE SolGel WAX column utilizing molecular polarities for separation. In this study, a 2D GC configuration (heartcut) was used to achieve better separation of the VOCs, where just a subset of the VOCs of interest were passed between the first and second columns within a specific retention time range through control of a heartcut valve between the first and second columns. The better separation achieved by using the 2D GC configuration enhances the VOC identification capability. In this study, the retention times of the heartcuts for the first column were set to 0–5, 5–10, 10–15, 15–20, and 20–25 minutes.

Thermal desorption of the analytes from the SPME fiber was achieved via manual injection into a split/splitless heated injection port in the splitless mode at 240°C. The inlet gas was provided by ultrahigh purity (UHP) helium in constant pressure mode at 9.6 psi. The oven temperature was held at 40°C for 3 minutes, then increased at a rate of 7°C/min to 105°C, subsequently increased at a rate of 4°C/min to 240°C, and then held at the latter temperature for 3 minutes. Backflush of the pre-column was activated between 46 and 50 minutes. The MS was set in electron ionization (EI) mode at 70 eV with a scanning rate of 4.51 scans/sec over the mass range of 35–350 amu, rounding to 1 amu resolution. The ion source and quadrupole temperatures were set at 230°C and 150°C, respectively.

The system automation and data acquisition software included a MultiTrax ver 8.01 from Microanalytics as well as a Chemstation ver. E.01.00.237 from Agilent. The National Institute of Standards and Technology (NIST) mass spectral search program version 2.0d was used for compound identification.

### Reagents

The gamma-butyrolactone (Cat#: B103608), potassium chloride (Cat#: P9541), and cycloheximide (Cat#: C4859) reagents used in this study were obtained from Sigma-Aldrich (St. Louis, MO).

### Fluorescent microscopy

The fluorescent images were recorded by an Olympus fluorescence microscope (IX71) linked with an Olympus DP71 digital camera. Exposure time was 1/6 second for all cells.

### Cell viability assays

The Cell Proliferation Kit XTT (Thermo Fisher Scientific, Cat#: X-6493) was used to measure cellular viability and cytotoxicity. The protocol was performed as described by the manufacturer. Briefly, cells were grown in a 96-well plate with or without tested compounds for two days, followed by the direct addition of 25 μL of XTT/PMS solution to each well, each containing a 100 μL cell culture. The cells were then incubated for 2 hours at 37°C in a CO_2_ incubator. Changes in cell viability were determined by measuring the absorbance at 450 nm using a Multiscan FC microplate reader (Thermo Fisher Scientific). Cell viability (Relative Viability) was calculated as a percent of control cells (non-treated).

### Electrophysiology

Differentiated LNCaP or Vero cells were visualized using a Nikon Eclipse-Ti inverted microscope (Nikon^®^, Nashua, NH, USA) equipped with Hoffman optics. Recordings were performed at room temperature (22–24°C). Recording electrodes were made from thin wall borosilicate glass (3–4 MΩ) using a two-step puller and filled with a solution consisting of 120mM KCl, 2mM MgCl_2_, 10mM HEPES-KOH, and 10mM EGTA, pH 7.4. The composition of the normal external saline was 145mM NaCl, 5.4mM KCl, 0.8mM MgCl_2_, 5.4mM CaCl_2_, and 13mM HEPES-NaOH, pH 7.4. Changes in the resting membrane potential were recorded in the current clamp configuration with I = 0 using a MultiClamp 700A amplifier and Pclamp software (Molecular Devices, USA). Pipette offset, whole cell capacitance, and series resistance (usually <10 MΩ) were compensated automatically with the MultiClamp 700B Commander. Sampling rates were between 5 and 10 kHz. Drugs were applied for 1–3 min with a ValveLink 8 perfusion system (AutoMate Scientific Inc., USA). Changes in membrane potential were recorded 2–3 min after application of the GBL, KCl, or the virus. Data was analyzed using Clampfit 10 (Molecular Devices, USA) and represented as mean ± SEM (standard error of the mean) with n representing the number of recorded cells (from at least two cell culture platings). Statistical comparisons were carried out using analysis of variance (ANOVA) (when multiple comparisons were involved) or the Student’s *t*-test (involving two sets of samples). A result of p≤0.05 was considered statistically significant.

### q-RTPCR and qPCR

Quantitative analyses of gene expression were performed by quantitative reverse transcription polymerase chain reaction (q-RT-PCR) using myiQ iScript One-Step RT-PCR kits from Bio-Rad (Cat#: 1725150). Experiments were performed in triplicate with one set of primers per reaction. The primer sequences for TK are 5’-GCA CGT CTT TAT CCT GGA TTA CG-3’ and 5’-TGG ACC ATC CCG GAG GTA AG-3’ with the Taqman probe 5’- /56-FAM/CCG GGA CGC CCT GCT GCA /3BHQ_1/-3’. The control housekeeping gene was peptidylprolyl isomerase A (PPIA) (5′-AGC ATA CGG GTC CTG GCA TCT-3′ and 5′-CAT GCT TGC CAT CCA ACC ACT CA-3′) and was reported to have expression without interference from HSV-1 infection [5]; The qRT-PCR reactions were carried out at 45°C for 10 minutes, 94°C for 2 min, and then 35 cycles of 94°C for 15s, 69°C for 15s, and 72°C for 15s.

The calculations of normalized gene expression and viral genome copy number were described previously [6]. In summary, we compared the Relative Quantity (RQ) of the tested sample and the RQ of the control. The RQ was first measured as RQ_Gene X_ = E_Gene X_^CT(Min) -CT(Sample)^ where E is the efficiency of the primer set and CT is the cycle of threshold. After getting the RQ of the tested sample and RQ of the control, the Normalized Relative Quantity (NRQ) was presented as: NRQ_Gene X_ = RQ _Gene X_ / RQ _Control_.

### Plaque assay

Cell monolayers were prepared in 384-well plates. Supernatant dilutions collected from infected cells were prepared, followed by incubation with a monolayer for 24 hours. The incubated monolayers were counted by automated high throughput imaging using a Cytation 3 Cell Imaging Multi-Mode Reader from Bio-Tek (Cat#: CYT3V, Winooski, VT, USA) with Gen5^™^ software (Cat#: Gen5). The protocol was conducted as previously described [7].

### Statistical analysis

Statistical analyses were performed by ANOVA (involving multiple sets of samples) with post-hoc Dunnett’s test. Student’s *t-*distribution was used while two set of samples were compared. Bars labeled with * were found to be statistically significant from each other with a p<0.05. The *p* value of greater than 0.05 was considered “not significant” (NS).

## Results

### Infection of Vero cells

The VOCs from the infected cells were collected via headspace sampling using a 50/30 μm Divinylbenzene/Carboxen Solid Phase MicroExtraction (SPME) device (Fig 1) followed by analysis using 2D GC/MS.

### 2D GC/MS analysis

At the end of the infection time period, a SPME was inserted into the headspace to collect VOCs followed by a 2D GC/MS analysis. The compound identification was performed by comparing the measured mass spectrum of a VOC against spectra in the NIST mass spectral library to find the best match. The identified compounds were further confirmed using a standard. When generating chromatograms for the same heartcut time window, the same SPME was used for headspace sampling to reduce systematic error.

#### Heartcut analysis: 0–5 min

**Fig 2:**
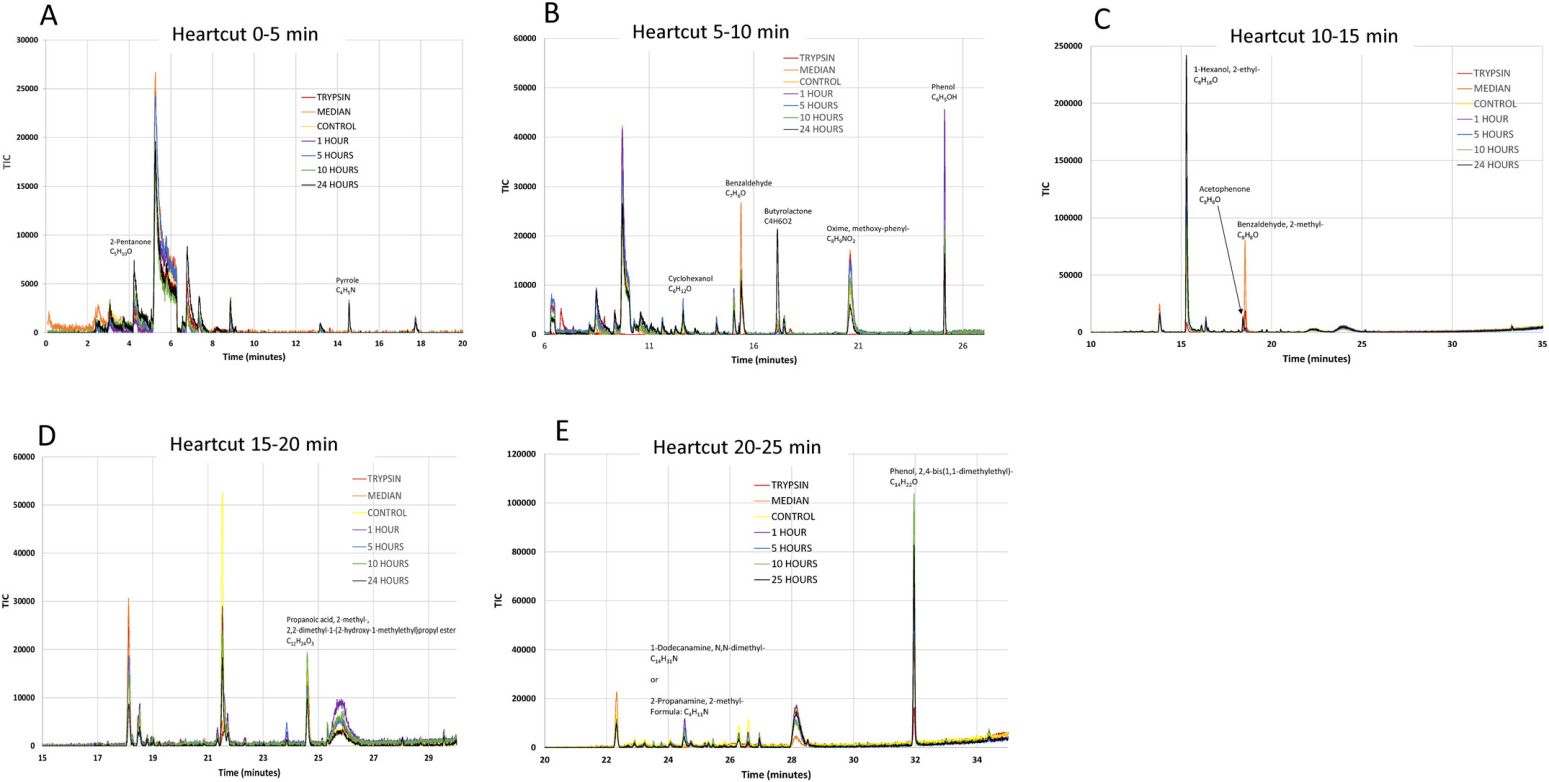
Overview of VOC characterization. The chromatograms were recorded using a 2D GC-GC configuration (heartcut) to attain better VOC separation prior to identification using a MS. The heartcut time periods logged are A. 0–5 min; B. 5–10 min; C. 10–15 min; D. 15–20 min; and E. 20–25 min.

For the chromatogram generated using the heartcut period of 0 to 5 minutes, the intensity of 2-Pentanone (C_5_H_10_O) was found to rise with increasing infection time (Fig 2A). At the retention time of 14.5 minutes, another peak that was identified as Pyrrole (C_4_H_5_N) was observed. It is noted that this peak was only detected in control and infection samples, but not from the controls such as trypsin or media samples (Fig 2A).

#### Heartcut analysis: 5–10 min

The chromatogram collected using the heartcut period of 5 to 10 minutes showed a small peak at 12.5 minutes, which was identified to be cyclohexanol (C_6_H_12_O). Several peaks were observed for 1 and 5 hpi samples (Fig 2B). At the retention time of 15.4 minutes a significant peak was identified as Benzaldehyde (C_7_H_6_O). This peak was very strong in the control and median samples but decreased in magnitude upon infection (Fig 2B).

**Fig 3:**
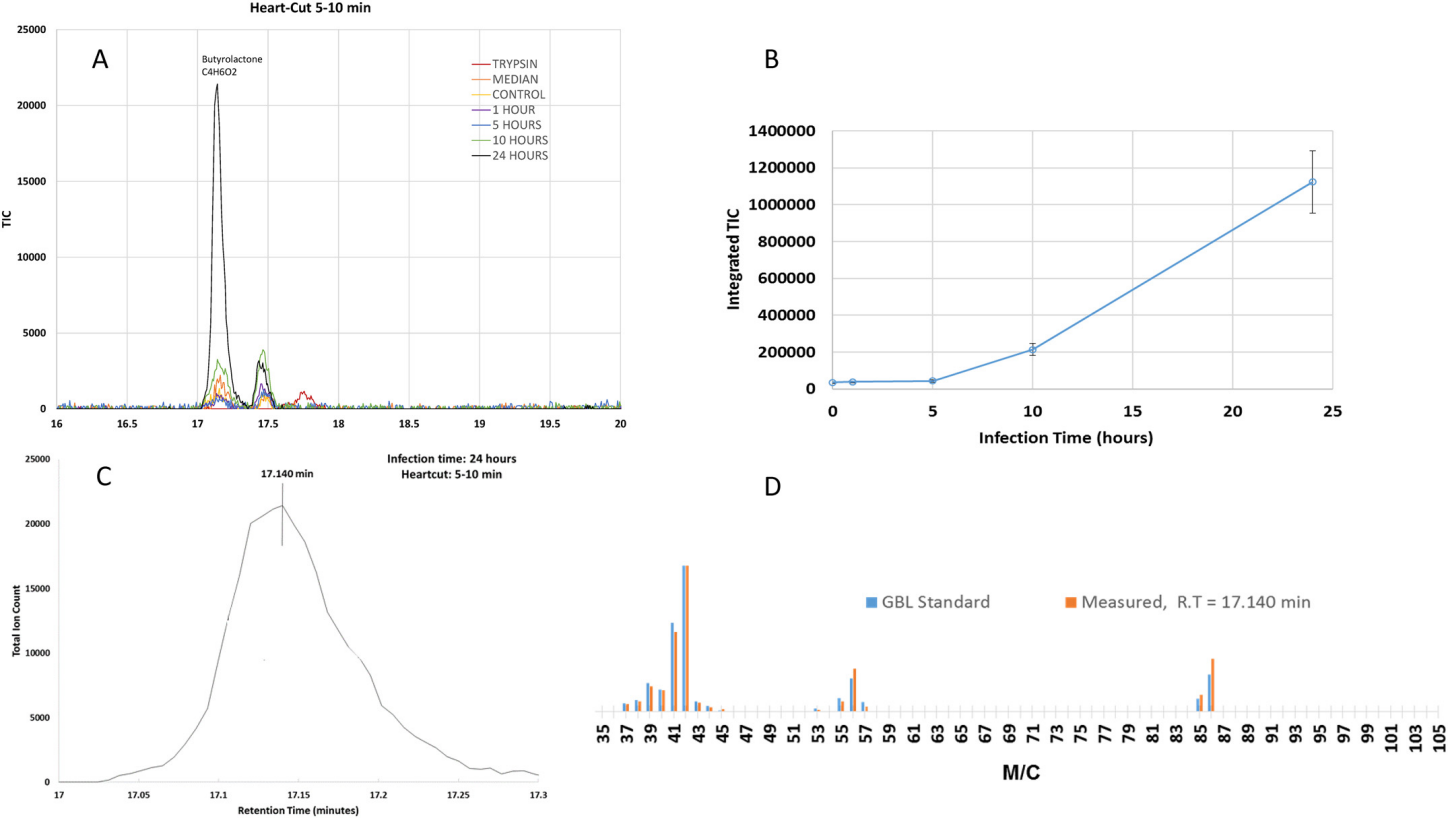
Examination of GBL release profile. A. The GBL peak was identified from the chromatograms taken using the 5–10 min heartcut. A close-up view shows the peak intensity was very weak among all samples and the controls except for the 24 hpi one, for which the GBL peak exhibited a very large increase. B. Measurement of integrated TIC during different infection times of 0, 1, 5, 10, 24 hpi, A significant increase was detected at 24 hpi. C. The enlarged GBL peak (labelled with retention time) where the measured mass spectra were compared with the GBL standard mass spectrum as shown in D. D. Comparison of mass spectra collected at different retention times with the GBL standard mass spectrum from the NIST library.

An interesting peak also occurred in this time period at 17.1 minutes with an intensity that increased to maximum value at 24 hpi (Fig 2B and a close-up view in Fig 3A). Almost no such peak was observed in any of the control samples, including trypsin, media, and the uninfected Vero Cells (Figs 2B and 3A).

In addition, peaks of Oxime and methoxy-phenyl- (C_8_H_9_NO_2_) were observed that exhibited a complex pattern in which the 24 hpi sample has the lowest peak intensity (Fig 2B). Finally, Phenol (C_6_H_5_OH) was also detected, with a peak spike for the 1 hpi sample (Fig 2B).

#### Heartcut analysis: 10–15 min

The only meaningful peak identified from the 10–15 minute heartcut analysis was 1-Hexanol, 2-ethyl- (C_8_H_18_O). The data shows that the 24 hpi specimen produced the highest peak intensity among all the samples (Fig 2C).

#### Heartcut analysis: 15–20 min

All samples produced a peak at 24.5 minutes of retention time, which was verified to be Propanoic acid, 2-methyl-, 2,2-dimethyl-1-(2-hydroxy-1-methylethyl)propyl ester (C_12_H_24_O_3_). However, the peak does not show any correlation with infection time. (Fig 2D).

#### Heartcut analysis: 20–25 min

The compound 1-Dodecanamine, N, N-dimethyl- (C_14_H_31_N) or 2-Propanamine, 2-methyl- (C_4_H_11_N) was detected from the chromatograms (Fig 2E). It is noted that the media, trypsin, and control samples did not have the peak for this compound, but the infected samples all had the peak, with the 1 hpi specimen showing the highest peak intensity.

### Characterization of GBL detected upon infection

Within the 5–10 minute heartcut periods, the GBL concentration from the cell pellet peaked at 17.1 minutes. The concentration increased beginning at 10 hpi infection and achieved a maximum at 24 hpi (Fig 3A and 3B). A mass spectra taken at 17.140 minute matched with the NIST standard mass spectrum of GBL (Fig 3C). We further repeated the GC/MS analysis using a GBL standard to confirm that the peak was GBL (Fig 3D).

### GBL detection from the infected culture media of Vero cells but not from Vero under stress

**Fig 4:**
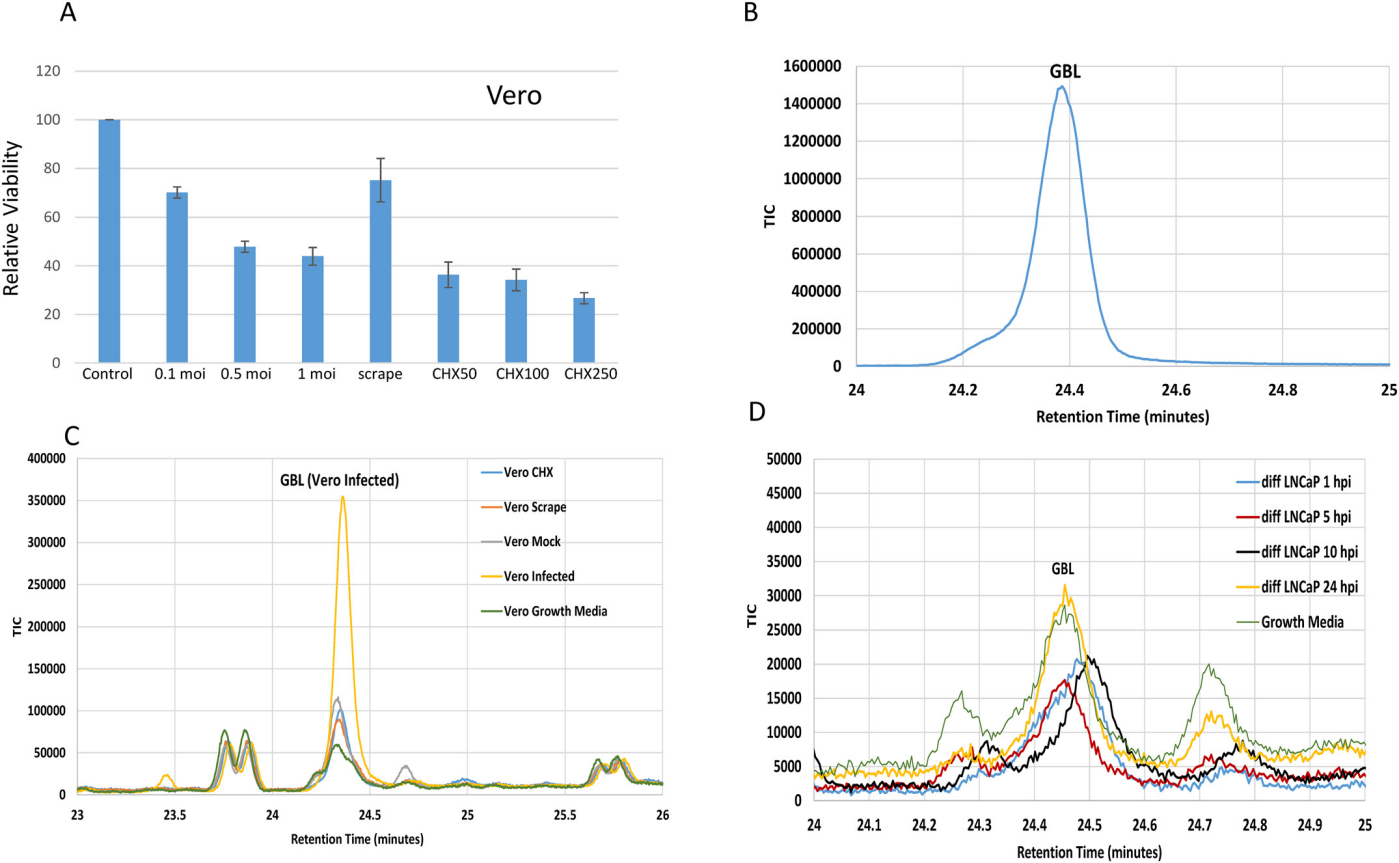
Characterization of GBL induction during stress condition and differentiated LNCaP cells. A. Relative viability of cells upon infections, scraping, and CHX treatment was evaluated in comparison to control. They were measured at 48 h post infection or post treatment. B. A GBL standard was run as control for GC/MS analysis. The spike was detected approximately at the retention time of 24.4 min. C. GBL was identified only in infected Vero cells but not from CHX, scraping, mock control, and culture media. The CHX concentration was 250 μg/ml. D. The GBL detection was not different in the infected LNCaP cells in comparison to the control. Infection of LNCaP was performed with the same moi of Vero infection.

To investigate whether the GBL production resulted from stress, Vero cells were either treated with CHX at a toxic concentration or scraped as a mechanical trauma for comparison against a sample from 24 hpi. The viability of the cells was tested via XTT assays (Materials and Methods) with different moi and different concentrations of CHX. It was found that infection at a moi of 1, scraping, and a CHX at 250 μg/ml resulted in cell deaths of 56%, 25%, and 73% in the samples in comparison to no treatment control, respectively (Fig 4A). The media were collected and subjected to SPME headspace sampling followed by GC/MS analyses to avoid freeze-thaw. A GBL standard was first run and peaked approximately at 24.4 minutes (Fig 4B). The results indicated that GBL can be detected from the infection sample of Vero cells but not from the cell samples subject to scraping, treatment with CHX (250 μg/ml), the media, or infection controls (Fig 4C). Additional similar studies using a differentiated LNCaP displayed no significant induction (Fig 4D), suggesting that the release of GBL was not detected from HSV-1 infection of a human neuron-like cells.

### GBL altered Resting Membrane Potential (RMP) of differentiated LNCaP

**Fig 5:**
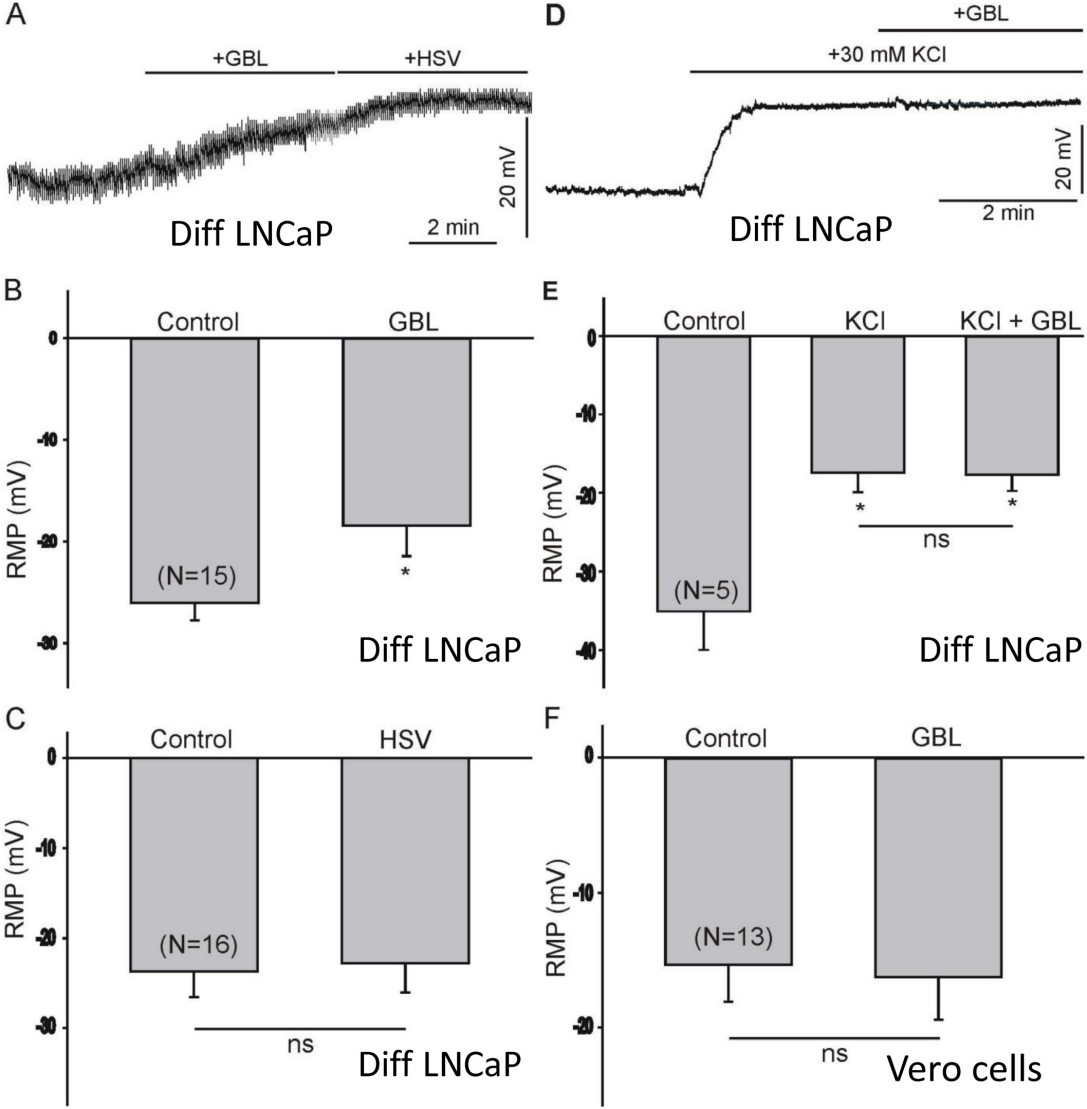
GBL altered RMP of differentiated LNCaP. A. Stimulation of differentiated LNCaP cells with 40 mM GBL caused a significant depolarization of the RMP. B. Treatment with 40 mM GBL for 2–3 min resulted in a significant depolarization of the RMP (p≤0.05 using single tail, unequal variance, unpaired student's *t*-test). In this and subsequent bar figures, N represents the number of recorded cells in each test group. C. Stimulation of differentiated LNCaP cells with HSV-1 did not result in any change in RMP by Student’s *t*-test (NS denotes No significance, *p>0*.*05*). D. Stimulation of differentiated LNCaP cells with 30 mM extracellular KCl resulted in a significant depolarization of the RMP. Exposure to GBL did not generate any further change in the RMP following membrane depolarization with 30 mM KCl. E. Summary of the changes in the RMP evoked by 30 mM KCl and GBL treatment in differentiated LNCaP cells. Bars labeled with an asterisk (*) were shown to be statistically different compared to controls with a p≤0.05 using an ANOVA multiple comparison test. NS indicates Not Significant. F. Stimulation of Vero cells with GBL (40 mM) has no significant effect on the RMP. Statistical study was calculated by the Student’s *t*-tests.

Since GBL production was significantly increased following HSV-1 infection, we tested the possible effect of this compound on the resting membrane potential of differentiated LNCaP cells. Changes in resting membrane potential (RMP) following stimulation with GBL were assessed in the current clamp configuration with I = 0. The RMP of the differentiated LNCaP cells was between -20 and -40 mV (average value = -28 ± 2 mV, n = 21). Stimulation of LNCaP cells with 40 mM GBL caused a significant depolarization of the RMP (~10 mV, Fig 5A and 5B). On the contrary, application of the virus did not have any noticeable effect on the RMP (Fig 5A and 5C). To investigate whether changes in the equilibrium potential for K^+^ ions can alter the effect of GBL on the RMP, cells were stimulated with 30 mM extracellular K^+^ ions, followed by stimulation with GBL. As shown in Fig 5D, application of 30 mM extracellular K^+^ ions caused a significant depolarization of the RMP. Application of GBL in the presence of 30 mM extracellular K^+^ ions did not result in any further membrane depolarization (Fig 5D and 5E). Thus, disruption of the K^+^ equilibrium potential prevents the depolarizing effect of GBL. Further study indicated that GBL had no effect on the RMP of Vero cells (Fig 5F).

### GBL decreased viral gene expression and replication in differentiated neuronal cells

**Fig 6:**
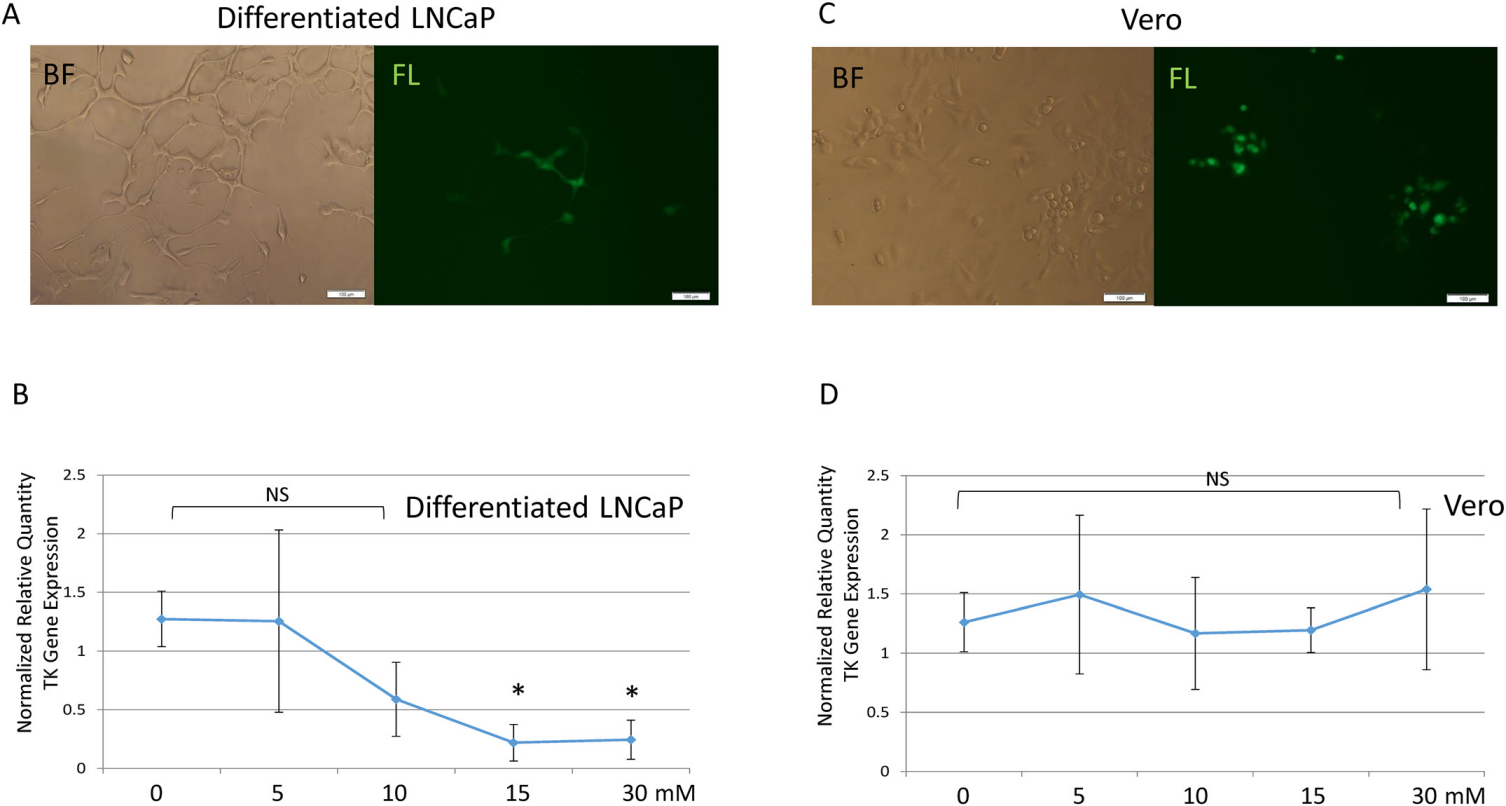
Concentration-dependent effects of GBL on viral gene expression. A. Fully differentiated LNCaP cells infected by recombinant HSV-1 emitting GFP. The image was captured with an exposure time of 1/6 sec. BF: Bright field; FL: Fluorescent microscopy. B. The concentration-dependent effects of GBL on infection of differentiated LNCaP was determined at 48 hpi by q-RT-PCR measuring HSV-1 TK standardized by the housekeeping gene PPIA. A statistical study was presented using ANOVA with Dunnett’s post-hoc test. Bars labeled with an asterisk (*) were found to be statistically different in comparison to a vehicle with a p<0.05. NS: Not Significant. C. Vero cells were infected by recombinant GFP expressing HSV-1. The exposure time was 1/6 sec when the image was captured. BF: Bright field; FL: Fluorescent microscopy. D. The concentration-dependent effects of GBL on infection of Vero was determined by q-RT-PCR using the same method described in Fig 6B. A statistical study was determined by ANOVA. NS: Not Significant with p>0.05.

HSV-1 can establish latency in neurons after primary acute infection [1]. It was of interest to study if GBL had a regulatory role in HSV-1 neuronal infection. To address this hypothesis, human neuron-like LNCaP cells were infected with HSV-1 (Fig 6A), followed by treatment of 5–30 mM of GBL 1h after the infection. These conditions were based on the toxicity analyses that indicated the TD_50_ of GBL for differentiated LNCaP is 218 mM (data not shown). Previous investigations indicated that this human neuron-like cells displayed suppressive effects to HSV-1 infection [3,6]. The regulatory effects of GBL on HSV-1 infection were first tested by q-RT-PCR at 48 hpi measuring HSV-1 thymidine kinase (TK) transcription that was normalized to the cellular gene PPIA, which was reported to not be affected by viral infection [5]. The results showed a concentration-dependent repression of HSV-1 gene expression, with an approximately 80% decrease being observed at the concentrations of 15 mM and 30 mM in comparison to the control (Fig 6B). For the contrary case, treatment of infected Vero cells (Fig 6C) with 5–30 mM GBL had no regulatory effect on viral gene expression (Fig 6D). This observation suggested that GBL generated a repressive effect on HSV-1 TK transcription, which may result from replication decline. It was not due to the blocking of entry into the cells since GBL was added at 1 h post-infection.

**Fig 7:**
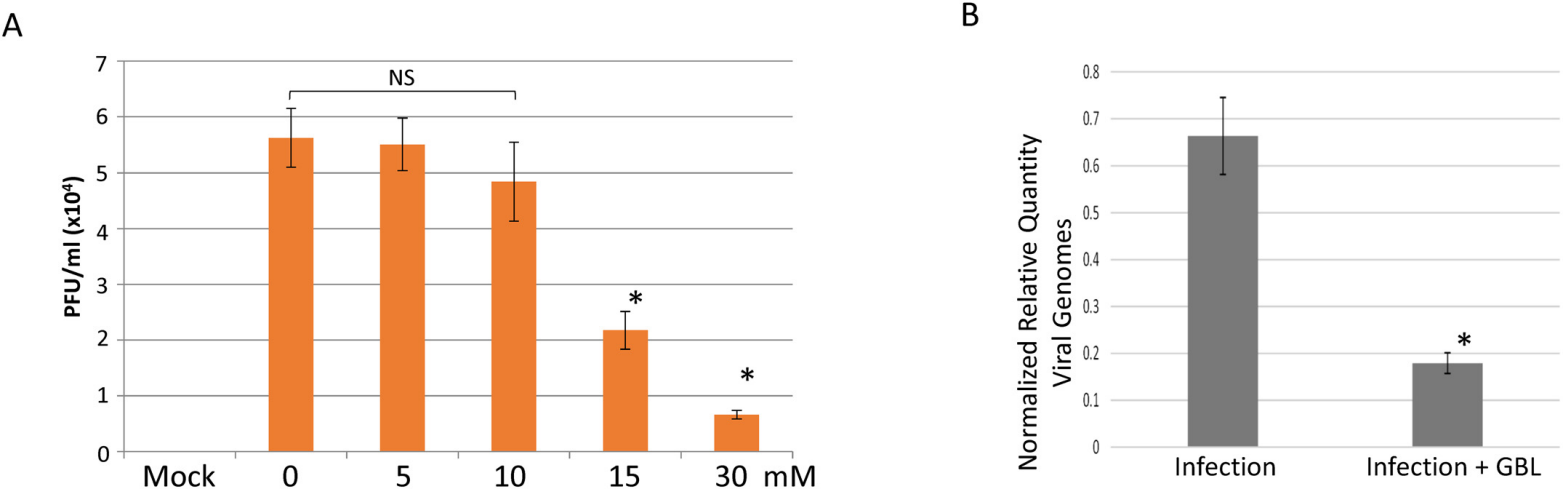
Concentration-dependent effects of GBL on viral replication. A. The replication was determined by plaque assays (PFU/ml) using media to measure the release of infectious virus particles. Different concentrations of GBL ranging from 5-30mM were used. A statistical examination was done by ANOVA with Dunnett’s post-hoc test. Bars marked with an asterisk (*) were shown to be statistically significant in comparison to no GBL control with a p<0.05. NS: not significant with p>0.05. B. Total DNA was isolated followed by a quantitative PCR measuring virus genome via the method of Normalized Relative Quantity (NRQ). A statistical investigation was executed using Student's *t*-tests. Bars marked with an asterisk (*) were shown to be statistically significant in comparison to the infection control with a p<0.05.

The GBL regulatory effects on viral replication were first addressed by plaque assays using media collected from the infection cultures at 48 hpi. It was observed that the control displayed a virus release approximately at 56,250 PFU/ml (Fig 7A). There was a concentration-dependent reduction of replication, with an approximately 88% decrease of infectious viral particles from the infected culture incubated with GBL at 30 mM (Fig 7A). The viral replication was further determined by quantitative PCR to measure virus genome copy changes due to GBL. The data indicated an approximately 73% decrease of HSV-1 genome copies in comparison to the control (Fig 7B). This observation supported the hypothesis that GBL may play a role in repressing viral gene expression and replication in neurons, suggesting a previously unknown inhibitory effect on viral infection of differentiated neuronal cells.

## Discussion

In this study, a GC/MS coupled with SPME headspace sampling was used to analyze VOCs from HSV-1 acute infection. The headspace absorption method by SPME is sensitive but has several limitations. The assumption of capture was that the concentration of VOCs in the headspace vapor detected by SPME is proportional to its concentration in the media, which would be released by the infected cells. It is likely that volatility can influence the capture and detection of a VOC. A SPME, depending on the coating materials, inevitably has minor preference when absorbing certain VOCs. In our experiment, a 50/30 μm Divinylbenzene/Carboxen^™^ on polydimethylsiloxane coating SPME was used, which adsorbs polar organic volatiles and semi-volatiles, with a carbon count between 3 and 20 and a molecular weight between 40 and 275. In terms of the releasing of the VOCs, differential emission should be minimal since the discharge temperature of the GC inlet is 240°C, which is high enough to release all adsorbed VOCs on the SPME. The SPME might also have a saturation limit since a small amount of the extracting phase associated with a solid support is placed in contact with the sample matrix for a predetermined amount of time. If the time is long enough, a concentration equilibrium can be established between the sample matrix and the extraction phase. When equilibrium conditions are reached, exposing the fiber for a longer amount of time does not accumulate more analytes. Another limitation is that compounds that are not stable at high temperatures could convert to other compounds at the GC inlet, which typically has a higher temperature (e.g. 240°C) to release adsorbed VOCs. Lastly, this is not an automatic or high throughput procedure since the machine allows only one heartcut window per run.

The detection of GBL was a novel discovery. The concentration of GBL in the headspace vapor is considered proportional to its concentration in the solution, which would be determined by the amount of GBL generated during the infection. When the SPME extraction is complete, the GBL concentration has reached distribution equilibrium between the headspace sample matrix and the SPME fiber coating. The mass of the GBL adsorbed by the SPME can be determined by the following equation [8]: n = (K_fs_V_f_V_s_C_0_)/(K_fs_V_f_+ V_s_), where n is the mass of analyte (GBL) extracted by the coating, K_fs_ is the fiber coating–sample matrix distribution constant, V_f_ is the fiber coating volume, V_s_ is the sample volume, and C_0_ is the concentration of GBL in the headspace. For the measurements of different infection samples, the same SPME was used, making K_fs_ and V_f_ constant. In addition, the sample volume and composition are the same for all infection samples, resulting in a constant V_s_ for measurements of different samples. Therefore, the mass of GBL collected by the SPME is determined by the concentration of GBL in the headspace vapor. Under the same GC/MS experimental conditions, the GBL peak area is proportional to the mass of GBL collected by the SPME. Therefore, the GBL peak area can be used to represent the GBL concentration in the infection sample.

As shown in Fig 3A, the GBL peak for the 24-hour infection time is significantly larger than those of other infection times and non-infected samples (including trypsin, media, and control samples). The GBL peak area was calculated by integrating the total ion count (TIC) under the peak for each sample. The dependence of the GBL peak area on the infection time is shown in Fig 3B. There is a slight increase in GBL concentration at the 10-hour infection time but a dramatic increase between 10 and 24 hours of infection time.

VOCs were first separated based on their boiling points and polarities, then identified using their mass spectra. Most of the identified VOCs have been studied for industrial purposes, but some have novel biochemical/biophysical roles that have yet to be well characterized. For example, the detection of GBL from mammalian cells upon infection from a human virus has never been reported before. GBL is known to be produced during stress in bacteria as an alternative metabolic pathway by GBL synthases [9,10]. In mammals it is reported that endogenous GBL can be physiologically detected in rat brains [11]. Although it is a naturally occurring component in some cases, as in humans, little is known regarding its physiologic roles.

We have detected a previously unknown effect of viral infection that results in the release of the volatile compound GBL. This compound possessed suppressive effects on viral replication in human neuron-like cells, specifically differentiated LNCaP cells. This discovery opens the possibility for studying the effects of HSV-1 evoked infection on differentiated cells using this cell line, with the many advantages it has to offer. First, it is a human cell line, not that of a mouse or rat. Second, it can be easily induced to full differentiation by androgen deprivation mimicking neurons [6,12–14]. Third, it expresses several important neuronal makers after differentiation, including ion channels [15–19]. Fourth, the PI3K pathway is active after differentiation, providing a very useful platform to study HSV-1 latency [3,20]. Nevertheless, there are several limitations regarding this model. For instance, it is an *in vitro* model and can’t reflect the real situations of lytic and latent infections. Differentiated LNCaP, although showing human neuron-like morphology and physiology, is not a true sensory neuron of trigeminal ganglia or dorsal root ganglia. HSV-1 replication, although reduced dramatically, never established a *bona fide* latency in this model.

It is not known how the virus triggered the production of GBL, but it is quite likely that the infectious process stimulates the production of Gamma-Hydroxybutyric acid or 4-hydroxybutanoic acid (GHB), which is then converted to GBL, since GHB and GBL have spontaneous inter-conversion [21,22]. In *Streptomyces*, GBL serves as a quorum sensing molecule [9,23–25]. Production of GBL is regulated and the intracellular concentration is positively co-related with the production of secondary metabolites such as antibiotics. Notably, GBL serves as a key signaling molecule that affects the expression of over 50 proteins and serves as a switch for primary to secondary metabolism [9]. The detailed mechanisms of GBL lactonization in mammalian cells are not clear, but may perhaps occur through lactonation or glucuronidation.

**Fig 8:**
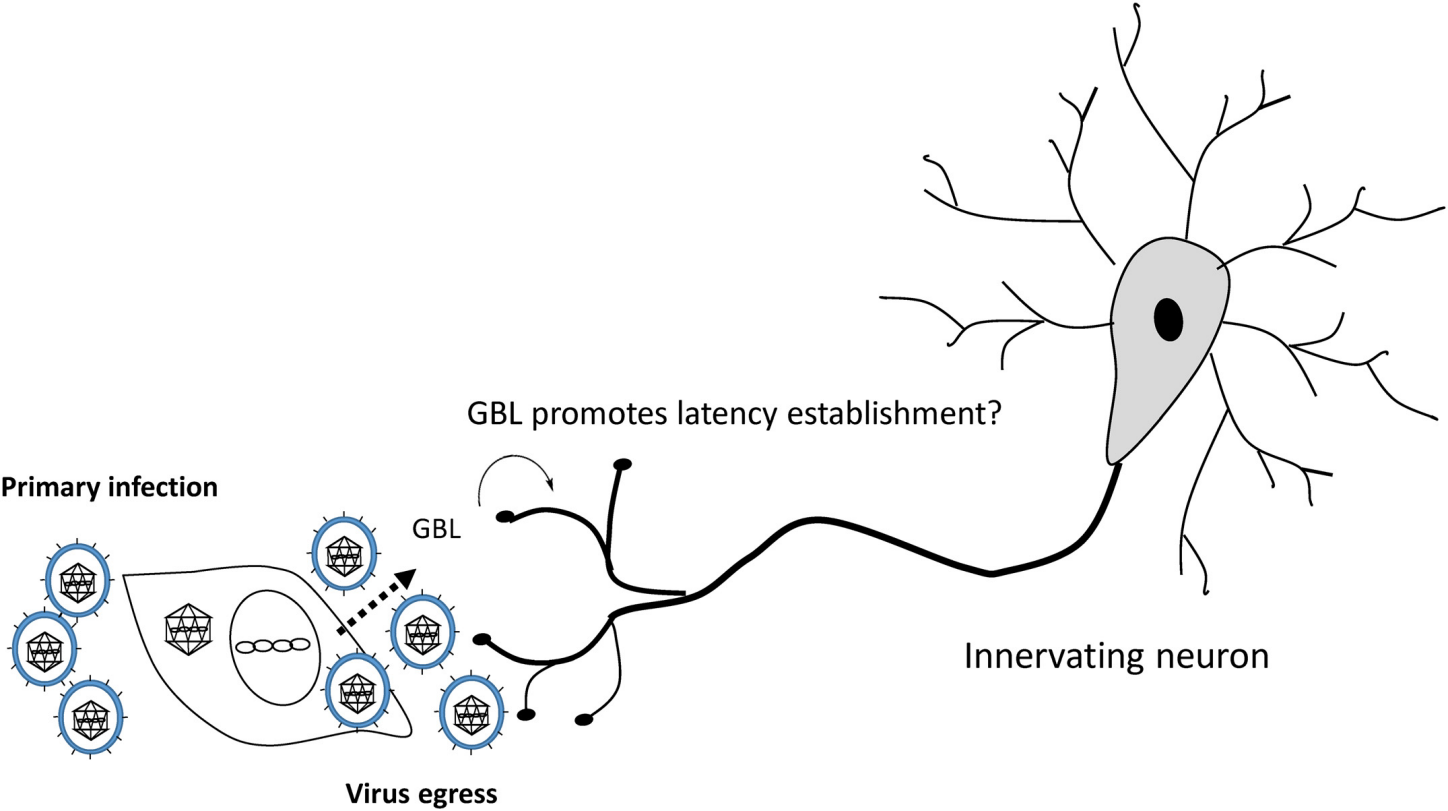
Proposed model of latency establishment by GBL. The working hypothesis is that GBL production is induced upon HSV-1 primary infection and this modulated the membrane potential of sensory neurons adjacent to the infected epithelial cells. This event can potentially alter the signaling pathway within the neurons, inhibiting viral gene expression and replication, thereby promoting latency establishment.

The hypothesis is that GBL released from infected cells may affect innervating neurons and interfere with subsequent viral replication. The putative roles of GBL in HSV-1 infection are unknown and remain to be elucidated. It is unclear how GBL repressed viral replication in differentiated cells. Our data suggested that the inhibitory effect of GBL on viral replication is likely mediated by its depolarizing effect on the membrane potential. The rationale is that it was previously shown that membrane depolarization by 30 mM KCl prevented viral replication in rat cerebral cortex neurons [26]. Current results were supportive of this hypothesis since GBL acted similarly to KCl in causing depolarization, and the depolarizing effect of GBL can be prevented by membrane depolarization with 30 mM extracellular K^+^. (Fig 5A). Additional investigation indicated that 30 mM KCl repressed HSV-1 replication in our model (data not shown). This observation may explain, at least in part, why GBL may have a role in promoting viral quiescence in neurons (Fig 8).

Our present results demonstrate that GBL causes a significant depolarization of the RMP in differentiated LNCaP cells, whereas HSV-1 stimulation has no effect. The molecular mechanism of GBL-evoked membrane depolarization in LNCaP cells remains to be clarified. Previous findings indicated that GBL can alter the conductance of several membranes, including the modulation of GABA_A_ receptors [27]. The inhibitory effect of GBL on viral replication is consistent with previous findings showing that membrane depolarization regulates HSV replication [26,28]. Thus, previous findings have demonstrated that membrane depolarization caused by increased extracellular K^+^ concentrations or inhibition of the Na^+^/K^+^ ATPase may lead to significant impairment of HSV replication in neuronal cells. This effect of membrane depolarization in preventing viral replication is primarily due to inhibition of viral early immediate genes [26]. It was reported that the inhibitory neurotransmitter GABA drastically promoted HSV-1 replication in primary neuronal cultures, and a GABA_A_ receptor antagonist totally eradicated the effects of GABA and reversed the activation [26]. This observation further suggested that action on the GABA_A_ receptor can modulate neuronal activity and control viral replication of HSV-1 in the nervous system, presumably during latency. A number of studies indicate that GABA_B_ receptors can repress the activity of adenylyl cyclase and diminish the conductance of neuronal cells to Ca^2+^ [29–31], which is very important for HSV-1 gene expression and replication [32,33]. In addition, GBL derivative exhibited neuro-protective properties and has been shown to defend cells from assaults such as hypoxia [34], another trigger of HSV replication [35–37]. However, the putative GBL effects on GABA receptors were complicated [27]. First, the experiments were done using a GBL derivative with modification/addition at the α or β position. Second, it appeared to have a dual modulating effect on the GABA_A_ receptor. In short, α-substituted alkyl lactones, more or less, potentiate GABA_A_ -mediated chloride currents, while compounds with β-substitution usually block GABA_A_ currents. Third, it is not always the case as described in the paper that some compounds can simultaneously decrease and increase GABA_A_ currents. Fourth, response times were also different, with some responses occurring quickly while others were slow. These authors actually proposed a model with two distinct GBL modulatory sites on the GABA_A_ receptor, an inhibitory “picrotoxin” site and an enhancing “lactone site”. Together, these studies indicate the possibility of a novel/complicated effect of GBL on HSV-1 biology.

In conclusion, the detection of GBL may assist in developing a new protocol that can be utilized in an electronic device for early diagnosis of HSV-1 infection, if this induction is specific to HSV-1. To achieve this goal, different viruses would need to be tested. Furthermore, it would be of interest to investigate using *in vivo* models to test if GBL released from primary HSV-1 infection can function as a neurotransmitter to modulate neuronal activity, thus serving as an aid in latency establishment in neurons. Moreover, it would not be surprising to discover that the GHB or GBL can be produced within neurons upon HSV-1 infection, thereby participating in latency establishment. More experiments are underway to explain this intriguing biochemical/biophysics incident.

## Abbreviations

HSV-1: Herpes Simplex Virus Type -1
VOCs: volatile organic compounds
SPME: Solid Phase Micro-Extraction
moi: Multiplicity of infection
GC/MS: Gas Chromatograph/ Mass Spectrometry
GABA: gamma-aminobutyric acid
GHB: Gamma-Hydroxybutyric acid or 4-hydroxybutanoic acid
GBL: Gamma-Butyrolactone
GPCR: G protein-coupled receptor
RMP: Resting Membrane Potential
CHX: Cycloheximide
